# Immediate gastrocnemius responses to intermittent cold compression at different pressure levels in soccer players

**DOI:** 10.1371/journal.pone.0361886

**Published:** 2026-11-05

**Authors:** Junjie Ma, Ying Liu, Fujian Sun, Changfei Guo, Can Gao, Dong Zhang, Dongxue Liang

**Affiliations:** 1 Institute of Artificial Intelligence in Sports, Beijing Key Laboratory of Interdisciplinary Intelligent Technologies in Sports Medicine and Engineering, Capital University of Physical Education and Sports, Beijing, China; 2 Sports Medical Supervision Research Center, Research Institute of Sports Medicine, General Administration of Sport of China, Beijing, China; Beijing Sport University, CHINA

## Abstract

**Background:**

Intermittent cold compression (ICC) is widely used for post-exercise recovery, but the optimal pressure for gastrocnemius recovery in soccer players remains unclear. This study investigated the immediate effects of different ICC pressure levels on gastrocnemius recovery after fatigue.

**Methods:**

Seven male collegiate soccer players (national Level 2 or above) participated in a randomized crossover trial. Following a 90-minute soccer-specific fatigue protocol, participants received a single 20-minute, 1°C ICC intervention at either low pressure (LP, 5–15 mmHg) or moderate pressure (MP, 5–50 mmHg), with a 48-hour washout. Outcomes included subjective perceptions (VAS pain, Borg CR10 fatigue), motor function (countermovement jump [CMJ] height, peak force, peak power, concentric work, rate of force development [RFD]), muscle mechanical properties (tone, stiffness, elasticity), and muscle architecture (thickness, pennation angle), assessed pre-and post-intervention.

**Results:**

Pain and RPE scores decreased significantly from pre- to post-intervention (p < 0.05), with no significant pressure or pressure × time effects. Significant time effects were found for CMJ height (F = 25.94, p = 0.002, η^2^ₚ = 0.812), peak force (F = 11.07, p = 0.016, η^2^ₚ = 0.649), and peak power (F = 13.17, p = 0.011, η^2^ₚ = 0.687), all declining post-intervention. Significant pressure × time interactions emerged for RFD (F = 6.07, p = 0.049, η^2^ₚ = 0.503) and lateral gastrocnemius logarithmic decrement (F = 9.21, p = 0.023, η^2^ₚ = 0.606).

**Conclusions:**

While both regimens alleviated subjective symptoms, significant pressure × time interactions were observed for RFD and lateral gastrocnemius logarithmic decrement, suggesting that compression pressure may influence the acute neuromuscular and mechanical responses to ICC. These findings should be interpreted cautiously given the small sample size and the absence of significant within-condition changes in RFD.

## Introduction

Soccer is a physically demanding intermittent sport characterized by continual low-intensity movement punctuated by frequent high-intensity sprinting, cutting, and directional changes during match play. These actions place substantial demands on the lower limb musculature, predisposing players to muscle fiber damage and transient neuromuscular function decline [1,2]. The gastrocnemius muscle, serving as the primary power source for ankle plantar flexion and rapid propulsion, is particularly susceptible to fatigue accumulation following high-intensity match play [3,4]. Therefore, identifying intervention strategies that can rapidly facilitate gastrocnemius functional recovery is of paramount importance, particularly within the context of congested fixture schedules.

Cryotherapy is a widely adopted physical intervention in sports recovery and rehabilitation, demonstrating efficacy in reducing inflammation and edema, alleviating pain, lowering tissue temperature, and modulating local hemodynamics [5,6]. To further enhance recovery efficiency, clinical practice has integrated cold stimulation with intermittent pneumatic compression, forming the intermittent cold compression (ICC) modality. This technique applies dynamic external pressure to directly promote local fluid circulation, synergizing with cold stimulation to produce analgesic effects and effectively accelerate soreness relief and microcirculatory improvement [7]. However, existing research has predominantly focused on proximal muscle groups such as the thigh, whereas the gastrocnemius, located in the distal lower limb, faces greater resistance to venous and lymphatic return, making the pro-reflux effect of external compression particularly critical.

The central controversy surrounding ICC intervention lies in the selection of optimal pressure parameters. Research indicates that introducing external mechanical pressure during cryotherapy produces additive effects superior to cryotherapy alone [8]; however, whether different pressures elicit differential immediate recovery effects remains a matter of debate [9]. In practical application, pressure parameter selection remains highly experience-dependent. Insufficient pressure fails to drive interstitial fluid clearance, whereas excessive pressure reduces comfort and may impede local microcirculatory perfusion. Thus, empirical comparison of the effects of different pressure levels carries substantial practical significance. Moreover, previous studies have largely been confined to single evaluation metrics such as pain scales or macroscopic motor performance [10,11], limiting the depth of insight into alterations in the intrinsic muscle state. The gastrocnemius, characterized by its relatively large pennation angle, possesses unique viscoelastic properties, and subtle changes in its internal stiffness, tension, and elasticity can significantly influence lower limb shock absorption and force production efficiency. More broadly, biomechanical assessment provides an important framework for linking changes in muscle mechanical and functional characteristics with athletic performance, injury-related loading, and rehabilitation outcomes. Integrating mechanical, architectural, and functional measures may therefore provide a more comprehensive characterization of post-exercise muscle status and support the development of evidence-based recovery and rehabilitation strategies [12].

To overcome the limitations of single-dimension assessments, the present study employed a randomized crossover design incorporating skeletal muscle ultrasonography and digitalized muscle tension assessment technology to compare the immediate recovery effects of low-pressure (LP) versus moderate-pressure (MP) ICC on the gastrocnemius following soccer-specific fatigue. Through a comprehensive four-dimensional evaluation encompassing subjective perception, motor function, muscle mechanical properties, and microstructural morphology, this study aimed to elucidate the specific therapeutic effects of different pressure conditions on analgesia, maintenance of lower limb explosive power, and improvement of deep muscle physical status, thereby providing evidence for the precise formulation of recovery strategies in sports practice.

## Materials and methods

### Participants

An a priori sample-size calculation was performed using G*Power version 3.1 before participant recruitment. The calculation used an F test for repeated-measures ANOVA (within factors), based on the four within-participant experimental measurements corresponding to the pre- and post-intervention assessments under the low- and moderate-pressure conditions. A large expected effect size of (f = 0.50) was assumed based on previously reported acute mechanical and functional responses to cryo-compression and related cooling interventions [13]. With an alpha level of 0.05, statistical power of 0.80, one group, four repeated measurements, an assumed correlation of 0.50 among repeated measurements, and a nonsphericity correction of 1.0, the required minimum sample size was seven participants.

Seven male collegiate soccer players classified at National Level 2 or above were recruited between [20 November 2025] and [20 December 2025]. Participant characteristics are presented in Table 1. The inclusion criteria were: (1) no history of lower-limb skeletal muscle injury within the preceding three months; and (2) no history of cold intolerance or cold allergy. The exclusion criteria were peripheral vascular dysfunction, lower-limb varicose veins or thrombotic disease, and any contraindication to the Game Ready system. Participants were withdrawn from the study if they experienced numbness or tingling, reported a visual analog scale (VAS) pain score greater than 8 during the experiment, or declined to continue participation. To minimize potential confounding factors, all participants were instructed to maintain their habitual diet and sleep patterns throughout the study period. Before each experimental session, participants were also required to refrain from any recovery intervention, including cryotherapy, heat therapy, massage, foam rolling, and static stretching.

**Table 1:** Participant characteristics (n = 7).

Age (years)	Height (cm)	Weight (kg)	Body fat (%)	Skeletal muscle mass (kg)	Body mass index (BMI, kg/m²)
22.3 ± 1.5	177.3 ± 3.6	75.2 ± 9.5	14.7 ± 6.9	36.2 ± 2.3	23.9 ± 2.8

Notes: Data are presented as mean ± standard deviation (SD). BMI, body mass index.

This study was conducted in strict accordance with the ethical principles of the Declaration of Helsinki. The experimental protocol was reviewed and approved by the Special Committee on Ethics and Morality of the Academic Committee of the Capital University of Physical Education and Sports (approval number: 2025A156). The researchers provided each participant with a thorough explanation of the experimental procedures and potential risks. All participants provided written informed consent and were explicitly informed of their right to withdraw from the study unconditionally at any time.

### Experimental design

This study employed a randomized crossover design. The intervention sequence was generated by a researcher not involved in data collection using a random number program. Prior to each experimental session, participants completed a 90-minute high-intensity soccer-specific exercise protocol on an outdoor football field. The protocol comprised two main components: 60 minutes of soccer-specific drill training and 30 minutes of simulated match-play training. The soccer-specific drill component included a 15-minute warm-up and activation period, 25 minutes of high-intensity technical training, and 20 minutes of repeated-sprint and change-of-direction exercises. The technical and conditioning drills incorporated sprinting, rapid deceleration and changes of direction, passing, dribbling, shooting, 1-v-1 actions, shuttle running, and repeated 20–30-m maximal sprints, with recovery periods of approximately 30–60 s between technical exercise bouts and approximately 1 minute between repeated sprints. The simulated match-play component consisted of 5v5 or 6v6 small-sided games, including goalkeepers, on a 30 × 40 m pitch, with three 7-minute playing bouts separated by 2-minute recovery periods. The remaining time consisted of standardized preparation and transition periods associated with the simulated match-play block. Players were instructed to maintain high-intensity running, active pressing, and rapid attacking–defensive transitions throughout the simulated match-play period. Bout duration is an important methodological characteristic of small-sided game protocols, as variations in playing duration may alter the accumulated exercise demands and technical responses. Previous research has shown that manipulating the number of players and training bout duration can modify mood-related and technical responses during soccer small-sided games [14]. Immediately after completion of the 90-minute protocol, perceived exertion was assessed using the Borg CR10 scale, and all participants reported RPE values of ≥8, indicating a high level of perceived exertion following the exercise protocol. The overall experimental procedure is shown in Fig 1.

**Fig 1:**
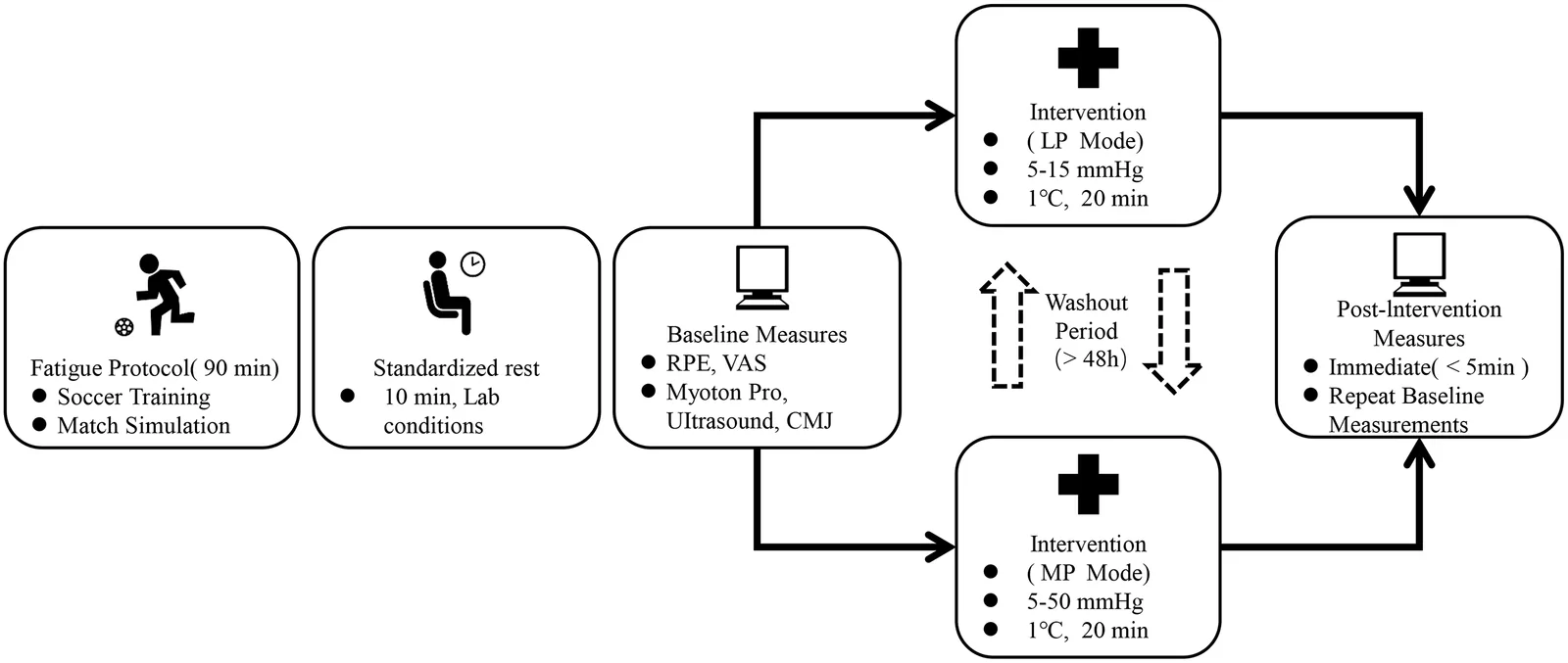
Experimental flowchart.

Pre-intervention measurement: Immediately upon completion of the training protocol, participants transferred to a temperature-controlled laboratory environment (22–24°C) and rested in a seated position for 10 minutes to eliminate physiological fluctuations induced by the transfer process. Following the rest period, baseline assessments of subjective fatigue perception and pain intensity were conducted using the 0–10 Borg CR10 scale and the 0–10 VAS, respectively. Subsequently, measurements were performed in the following order: skeletal muscle ultrasound imaging, gastrocnemius mechanical property assessment, and countermovement jump (CMJ) testing. This measurement sequence was designed to prevent alterations in muscle microstructure or fatigue accumulation induced by CMJ testing from compromising the accuracy of ultrasound imaging and muscle mechanical data.

Experimental intervention: Immediately following pre-intervention measurements, the ICC intervention was administered using a Game Ready GRPro 2.1 system. The intervention protocol was based on commonly used protocols in previous recovery research [11]. To maintain strict control over experimental variables, the target circulating-fluid temperature of the Game Ready GRPro 2.1 system was set at 1°C, and the duration of each intervention session was standardized at 20 minutes. This temperature represented the device setting for the circulating fluid rather than the actual temperature of the skin or underlying muscle tissue. Previous research using the same Game Ready system has demonstrated that the device temperature setting does not directly correspond to skin temperature. Throughout the ICC intervention, participants maintained a standardized prone position to control for the confounding effects of posture and gravity on distal lower limb venous return. Two pressure regimens were compared: low pressure (LP, 5–15 mmHg) and moderate pressure (MP, 5–50 mmHg). A minimum 48-hour washout period was implemented between experimental sessions to reduce the potential for carryover effects.

Post-intervention measurement: Post-intervention assessments were initiated immediately after completion of the ICC intervention and were performed in the same standardized order as the pre-intervention assessments: skeletal muscle ultrasound imaging, gastrocnemius mechanical property assessment, and CMJ testing. Owing to the short acquisition time required for the ultrasound and Myoton PRO assessments, the first CMJ trial was initiated approximately 3 minutes after removal of the cooling device. The same assessment sequence and approximate timing were maintained for all participants under both pressure conditions. To ensure objectivity of the data, all measurements were independently performed by two trained research personnel.

### Outcome measures and data collection

The experimental setup and data collection procedures are illustrated in Fig 2.

**Fig 2:**
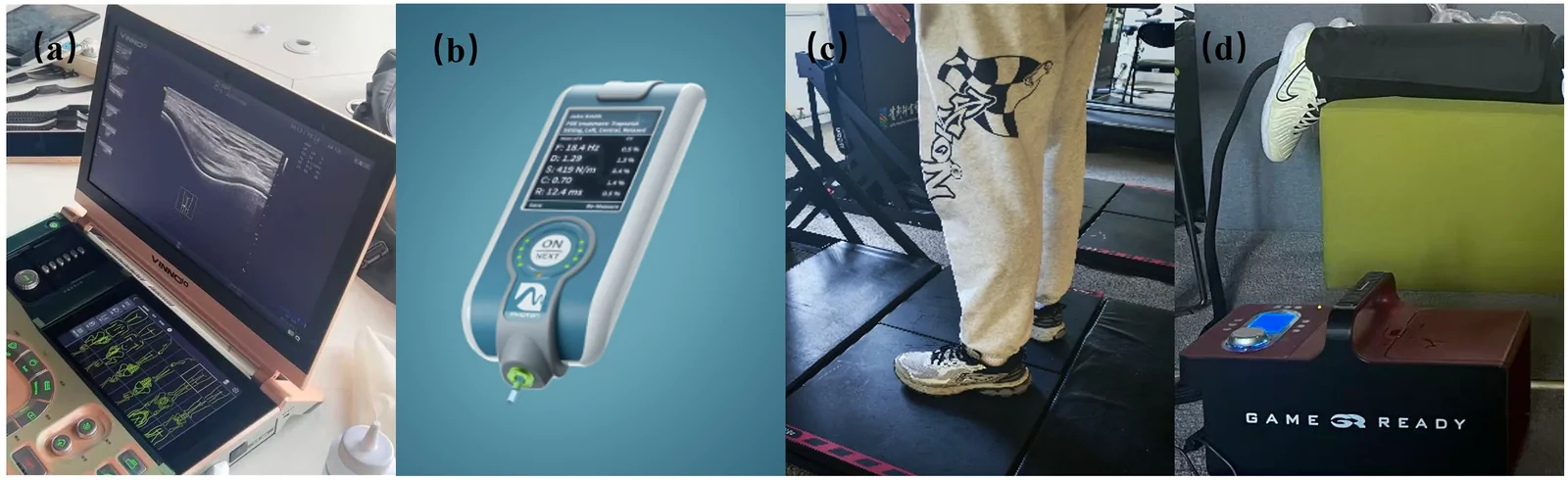
Experimental setup and data collection procedures. Note: Representative images of the equipment and procedures used in the study: (a) B-mode ultrasound imaging for assessment of gastrocnemius muscle architecture; (b) MyotonPRO digital palpation device for assessment of muscle mechanical properties; (c) countermovement jump (CMJ) testing for assessment of lower-limb functional performance; and (d) intermittent cold compression intervention using the Game Ready system.

#### (1) Body composition analysis.

Baseline morphological data were collected using an InBody 770 device (Korea). Measurements were uniformly performed in the morning under fasting conditions. Participants stood barefoot on the device electrodes for assessment. Key recorded variables included body mass, skeletal muscle mass, and body fat percentage.

#### (2) Motor function assessment.

CMJ performance was assessed using an AfaScan 100 fitness assessment system (Scanleader Technologies Co. Ltd., Santa Clara, CA, USA) equipped with a high-range force platform operating at a sampling frequency of 200 Hz. Participants performed CMJ with hands placed on hips, squatting rapidly upon verbal command followed by maximal vertical effort. Jump height, peak force, peak power, concentric work, and rate of force development (RFD) were automatically derived by the proprietary AfaScan 100 software from the force-platform data; no investigator-defined equations were applied to derive these outcomes. Each participant completed three trials, with a 30s passive recovery interval between consecutive trials, and the trial with the highest jump height was selected for analysis.

#### (3) Muscle mechanical properties.

The Myoton PRO (Myoton AS, Estonia) digitalized muscle tension assessment system was used for biomechanical testing of the gastrocnemius. Participants assumed a standardized prone position with both lower limbs naturally extended and relaxed. Measurement sites were selected at the most prominent belly of the gastrocnemius medial and lateral heads, and the skin was marked with a medical marker to ensure consistent positioning before and after the intervention. Prior to formal data collection, the sensor probe was vertically calibrated and placed perpendicular to the skin surface. Three consecutive measurements were taken at each site, and the mean value was used for analysis. The logarithmic decrement was used to represent muscle elasticity, reflecting the damping characteristics associated with mechanical energy dissipation over one oscillation cycle. Previous research has demonstrated that the logarithmic decrement is negatively correlated with muscle elasticity, with lower values indicating less mechanical energy dissipation and greater capacity of the muscle to return to its original shape [15]. Muscle stiffness was used to represent the muscle’s resistance to external compressive deformation, with higher values indicating greater stiffness. Muscle tone, represented by oscillation frequency, reflected the intrinsic tension of the muscle at rest, with higher frequencies indicating greater resting tension.

#### (4) Skeletal muscle architecture assessment.

Longitudinal scans of the gastrocnemius were performed using a VINNO 10 B-mode ultrasound diagnostic system (Feiyinuo Technology, Suzhou, China). During image acquisition, participants maintained a prone position with the knee and ankle joints in neutral alignment. Sufficient medical ultrasound gel was applied to the same marked sites used for Myoton PRO measurements. The operator positioned the ultrasound transducer perpendicular to the skin surface and parallel to the fascicle orientation. Throughout the procedure, transducer pressure was strictly controlled to prevent soft tissue compression, ensuring acquisition of clear longitudinal muscle fascicle images. Quantitative analysis of ultrasound images was performed using ImageJ software. Muscle thickness was defined as the shortest perpendicular distance between the superficial and deep aponeuroses. Pennation angle was defined as the angle formed between clearly visible muscle fascicles and the deep aponeurosis. All measurements were performed by a single image analyst who had received formal training in B-mode ultrasound and was blinded to the intervention condition. For each ultrasound image, muscle thickness and pennation angle were measured at three different regions, and the mean value was calculated for subsequent statistical analysis.

### Statistical analysis

All statistical analyses were performed using R language (version 4.5.0, R Foundation for Statistical Computing, Austria). Prior to inferential analysis, normality of the model residuals was assessed using the Shapiro–Wilk test. Residuals were normally distributed for all outcomes except concentric work and RPE. For these two variables, complementary Wilcoxon signed-rank analyses were performed on the corresponding within-participant contrasts. Because both within-subject factors comprised only two levels (pressure: LP vs. MP; time: pre-intervention vs. post-intervention), the assumption of sphericity was inherently satisfied and Mauchly’s test was not applicable. Two-way repeated measures analysis of variance (ANOVA) was used to evaluate the main effects and interaction of pressure condition (LP vs. MP) and measurement time (pre-intervention vs. post-intervention) on all outcome variables. When a significant pressure × time interaction was detected, Bonferroni-corrected post-hoc comparisons were conducted. Statistical results are reported as F-values, p-values, and partial eta-squared (η^2^ₚ) values. Partial eta-squared values were treated as descriptive measures of effect magnitude and were not used to infer effects when the corresponding hypothesis tests were not statistically significant. Two-sided 95% confidence intervals (CIs) were calculated for partial eta-squared (η^2^ₚ) estimates. The significance level was set at α = 0.05.

## Results

### Muscle mechanical properties

Results of the two-way repeated-measures ANOVA for muscle mechanical properties are presented in Table 2, and individual pre-to-post responses are shown in Fig 3. No significant main effects of time or pressure, nor their interaction, were observed for gastrocnemius tone (frequency) or stiffness (*p*>0.05). For logarithmic decrement, which is inversely related to muscle elasticity, significant main effects of time were found for both the medial head (*F*=13.97, *p*=0.010, *η*^2^ₚ=0.700) and lateral head (*F*=8.25, *p*=0.028, *η*^2^ₚ=0.579). A significant pressure × time interaction was observed for lateral head logarithmic decrement (*F*=9.21, *p*=0.023, *η*^2^*ₚ*=0.606). Bonferroni post-hoc analysis revealed that following LP intervention, the lateral head logarithmic decrement significantly increased compared to pre-intervention values (*p*=0.004), whereas no significant change was observed following MP intervention (*p*=0.668).

**Table 2:** Two-way repeated-measures ANOVA results for gastrocnemius mechanical properties.

Site	Variable	Effect	*F* (1,6)	*p*	*η²ₚ*	*95% CI*
	Frequency (Hz)	Pressure	0.12	0.745	0.019	[0.00, 0.45]
		Time	0.42	0.543	0.065	[0.00, 0.53]
		P × T	0.48	0.515	0.074	[0.00, 0.54]
Medial	Stiffness (N/m)	Pressure	0.01	0.944	0.001	[0.00, 0.21]
		Time	0.51	0.502	0.078	[0.00, 0.54]
		P × T	0.04	0.842	0.007	[0.00, 0.38]
	Logarithmic decrement	Pressure	4.24	0.085	0.414	[0.00, 0.75]
		Time	13.97	0.010	0.700	[0.10, 0.88]
		P × T	2.40	0.172	0.286	[0.00, 0.69]
	Frequency (Hz)	Pressure	2.59	0.159	0.301	[0.00, 0.70]
		Time	2.49	0.166	0.293	[0.00, 0.69]
		P × T	1.12	0.330	0.157	[0.00, 0.61]
Lateral	Stiffness (N/m)	Pressure	1.68	0.242	0.219	[0.00, 0.65]
		Time	5.29	0.061	0.469	[0.00, 0.78]
		P × T	1.73	0.236	0.224	[0.00, 0.65]
	Logarithmic decrement	Pressure	2.49	0.165	0.294	[0.00, 0.69]
		Time	8.25	0.028	0.579	[0.00, 0.83]
		P × T	9.21	0.023	0.606	[0.01, 0.84]

Notes: F values are reported with 1 and 6 degrees of freedom. Pressure represents the comparison between the low-pressure and moderate-pressure conditions; time represents the comparison between pre-intervention and post-intervention measurements; pressure × time represents the interaction between pressure condition and measurement time. η^2^p, partial eta squared; CI, confidence interval; LP, low pressure; MP, moderate pressure. Logarithmic decrement is inversely related to muscle elasticity, with lower values indicating greater elasticity.

**Fig 3:**
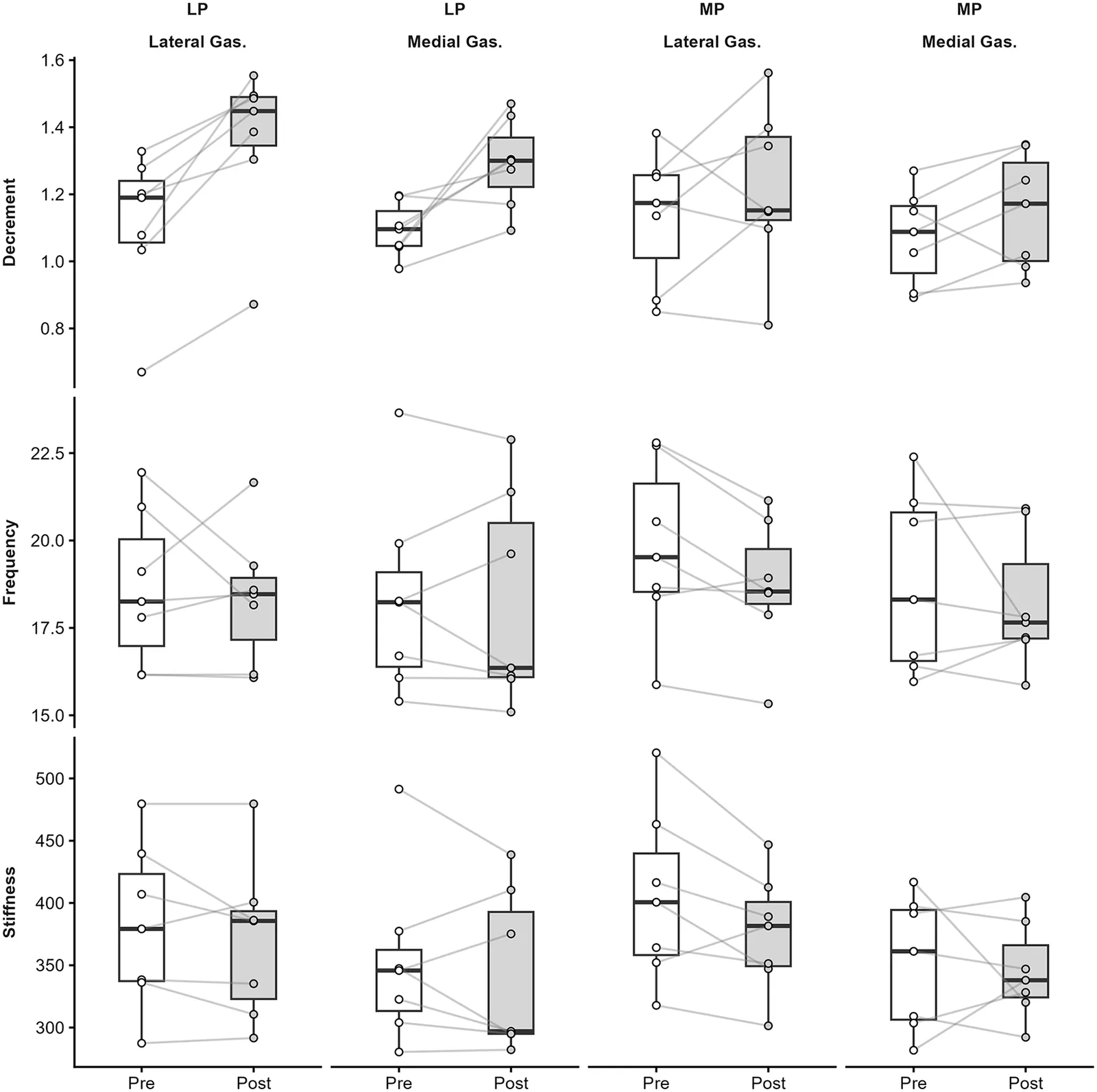
Gastrocnemius mechanical properties before and after intermittent cold compression. Note: The panels display oscillation frequency, stiffness, and logarithmic decrement of the medial and lateral gastrocnemius under low-pressure (LP) and moderate-pressure (MP) conditions. Open circles represent individual participants (n = 7), and connecting lines indicate paired pre-to-post changes. Boxplots represent the group distribution as the median and interquartile range. White and gray boxes indicate pre-intervention and post-intervention measurements, respectively.

### Motor function performance

Results of the two-way repeated-measures ANOVA for motor function are presented in Table 3, and individual pre-to-post responses are shown in Fig 4. Significant main effects of time were found for CMJ height (*F*=25.94, *p*=0.002, *η*^2^*ₚ*=0.812), peak force (*F*=11.07, *p*=0.016, *η*^2^*ₚ*=0.649), and peak power (*F*=13.17, *p*=0.011, *η*^2^*ₚ*=0.687), with all variables demonstrating significant decreases from pre- to post-intervention. No significant main effects of pressure or pressure × time interactions were observed for these variables (*p*>0.05). Concentric work showed no significant main effects or interaction (*p*>0.05).Because the residuals for concentric work deviated from normality, complementary Wilcoxon signed-rank analyses were performed, which likewise showed no significant pressure, time, or pressure × time effects (all *p*>0.05). A significant pressure × time interaction was detected for RFD (*F*=6.07, *p*=0.049, *η*^2^*ₚ*=0.503). Bonferroni post-hoc analysis indicated that neither LP nor MP intervention produced significant pre-to-post changes in RFD (LP: p=0.676; MP: p=0.308). However, the significant interaction indicated that the pattern of pre-to-post change in RFD differed between the two pressure conditions.

**Table 3:** Two-way repeated-measures ANOVA results for countermovement jump performance.

Variable	Effect	*F* (1,6)	*p*	*η²ₚ*	*95% CI*
CMJ height (cm)	Pressure	0.24	0.639	0.039	[0.00, 0.49]
	Time	25.94	0.002	0.812	[0.30, 0.92]
	P × T	1.07	0.341	0.151	[0.00, 0.61]
Peak force (N)	Pressure	0.09	0.780	0.014	[0.00, 0.43]
	Time	11.07	0.016	0.649	[0.04, 0.85]
	P × T	0.07	0.794	0.012	[0.00, 0.42]
Peak power (W)	Pressure	0.35	0.574	0.056	[0.00, 0.52]
	Time	13.17	0.011	0.687	[0.08, 0.87]
	P × T	0.26	0.628	0.042	[0.00, 0.50]
Concentric work (J)	Pressure	0.42	0.539	0.066	[0.00, 0.53]
	Time	0.69	0.437	0.104	[0.00, 0.57]
	P × T	0.06	0.821	0.009	[0.00, 0.40]
RFD (N/s)	Pressure	0.20	0.667	0.033	[0.00, 0.48]
	Time	1.02	0.351	0.146	[0.00, 0.60]
	P × T	6.07	0.049	0.503	[0.00, 0.79]

Notes: F values are reported with 1 and 6 degrees of freedom. Pressure represents the comparison between the low-pressure and moderate-pressure conditions; time represents the comparison between pre-intervention and post-intervention measurements; pressure × time represents the interaction between pressure condition and measurement time. η^2^p, partial eta squared; CI, confidence interval; CMJ, countermovement jump; RFD, rate of force development; LP, low pressure; MP, moderate pressure.

**Fig 4:**
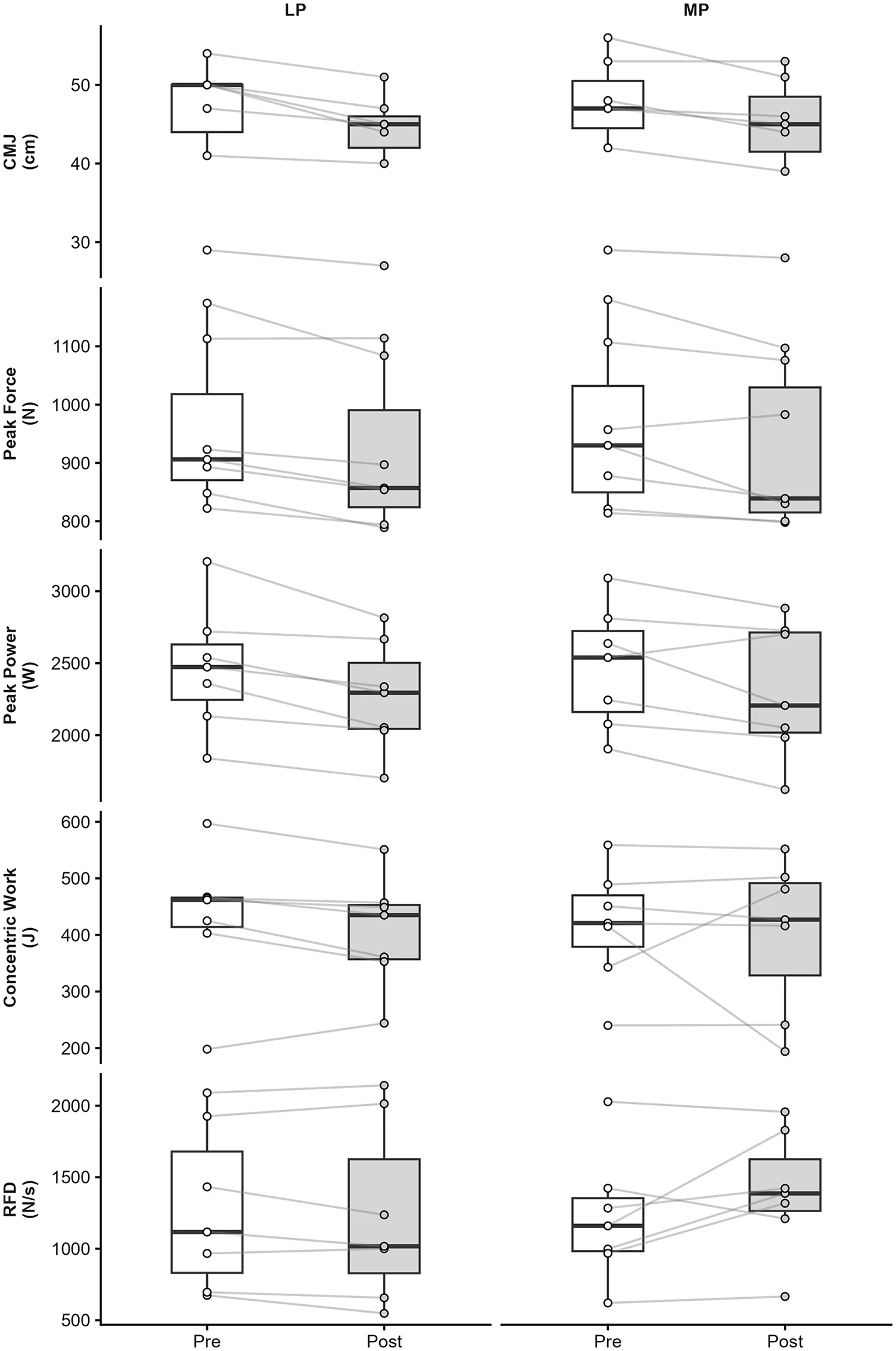
Countermovement jump performance before and after intermittent cold compression. Note: The panels display countermovement jump (CMJ) height, peak force, peak power, concentric work, and rate of force development (RFD) under low-pressure (LP) and moderate-pressure (MP) conditions. Open circles represent individual participants (n = 7), and connecting lines indicate paired pre-to-post changes. Boxplots represent the group distribution as the median and interquartile range. White and gray boxes indicate pre-intervention and post-intervention measurements, respectively.

### Muscle architecture

Results for muscle architecture are presented in Table 4. No significant main effects of time or pressure, nor pressure × time interaction, were observed for muscle thickness or pennation angle of either the medial or lateral gastrocnemius head (all *p*>0.05).

**Table 4:** Two-way repeated-measures ANOVA results for gastrocnemius muscle architecture.

Site	Variable	Effect	*F* (1,6)	*p*	*η²ₚ*	*95% CI*
	Muscle thickness (cm)	Pressure	0.04	0.845	0.007	[0.00, 0.38]
		Time	0.51	0.501	0.079	[0.00, 0.54]
Medial		P × T	0.37	0.568	0.057	[0.00, 0.52]
	Pennation angle (°)	Pressure	0.04	0.839	0.007	[0.00, 0.39]
		Time	0.49	0.511	0.075	[0.00, 0.54]
		P × T	1.05	0.345	0.149	[0.00, 0.60]
	Muscle thickness (cm)	Pressure	0.08	0.791	0.013	[0.00, 0.42]
		Time	0.08	0.787	0.013	[0.00, 0.42]
Lateral		P × T	1.37	0.285	0.186	[0.00, 0.63]
	Pennation angle (°)	Pressure	0.07	0.803	0.011	[0.00, 0.41]
		Time	0.64	0.453	0.097	[0.00, 0.56]
		P × T	0.46	0.524	0.071	[0.00, 0.54]

Notes: F values are reported with 1 and 6 degrees of freedom. Pressure represents the comparison between the low-pressure and moderate-pressure conditions; time represents the comparison between pre-intervention and post-intervention measurements; pressure × time represents the interaction between pressure condition and measurement time. η^2^p, partial eta squared; CI, confidence interval; LP, low pressure; MP, moderate pressure.

### Subjective perception

Results for subjective perception are presented in Table 5, and individual pre-to-post responses are shown in Fig 5. Significant main effects of time were found for both VAS pain scores (*F*=105.12, *p*<0.001, *η*^2^*ₚ*=0.946) and Borg CR10 fatigue ratings (*F*=9.63, *p*=0.021, *η*^2^*ₚ*=0.616), with both scores decreasing significantly from pre- to post-intervention. No significant main effects of pressure or pressure × time interactions were observed for either subjective measure (*p*>0.05). Because the residuals for RPE deviated from normality, a complementary Wilcoxon signed-rank analysis was performed. The time effect remained significant (*p*=0.016), whereas the pressure and pressure × time effects remained non-significant (both *p*>0.05), consistent with the repeated-measures ANOVA.

**Table 5:** Two-way repeated-measures ANOVA results for subjective fatigue and pain ratings.

Variable	Effect	*F* (1,6)	*p*	*η²ₚ*	*95% CI*
VAS	Pressure	0.18	0.682	0.030	[0.00, 0.48]
	Time	105.12	<0.001	0.946	[0.75, 0.98]
	P × T	1.35	0.289	0.184	[0.00, 0.63]
RPE	Pressure	0.06	0.818	0.010	[0.00, 0.40]
	Time	9.63	0.021	0.616	[0.02, 0.84]
	P × T	0.30	0.604	0.048	[0.00, 0.51]

Note: F values are reported with 1 and 6 degrees of freedom. Pressure represents the comparison between the low-pressure and moderate-pressure conditions; time represents the comparison between pre-intervention and post-intervention measurements; pressure × time represents the interaction between pressure condition and measurement time. η²p, partial eta squared; CI, confidence interval; VAS, visual analog scale; RPE, rating of perceived exertion; LP, low pressure; MP, moderate pressure.

**Fig 5:**
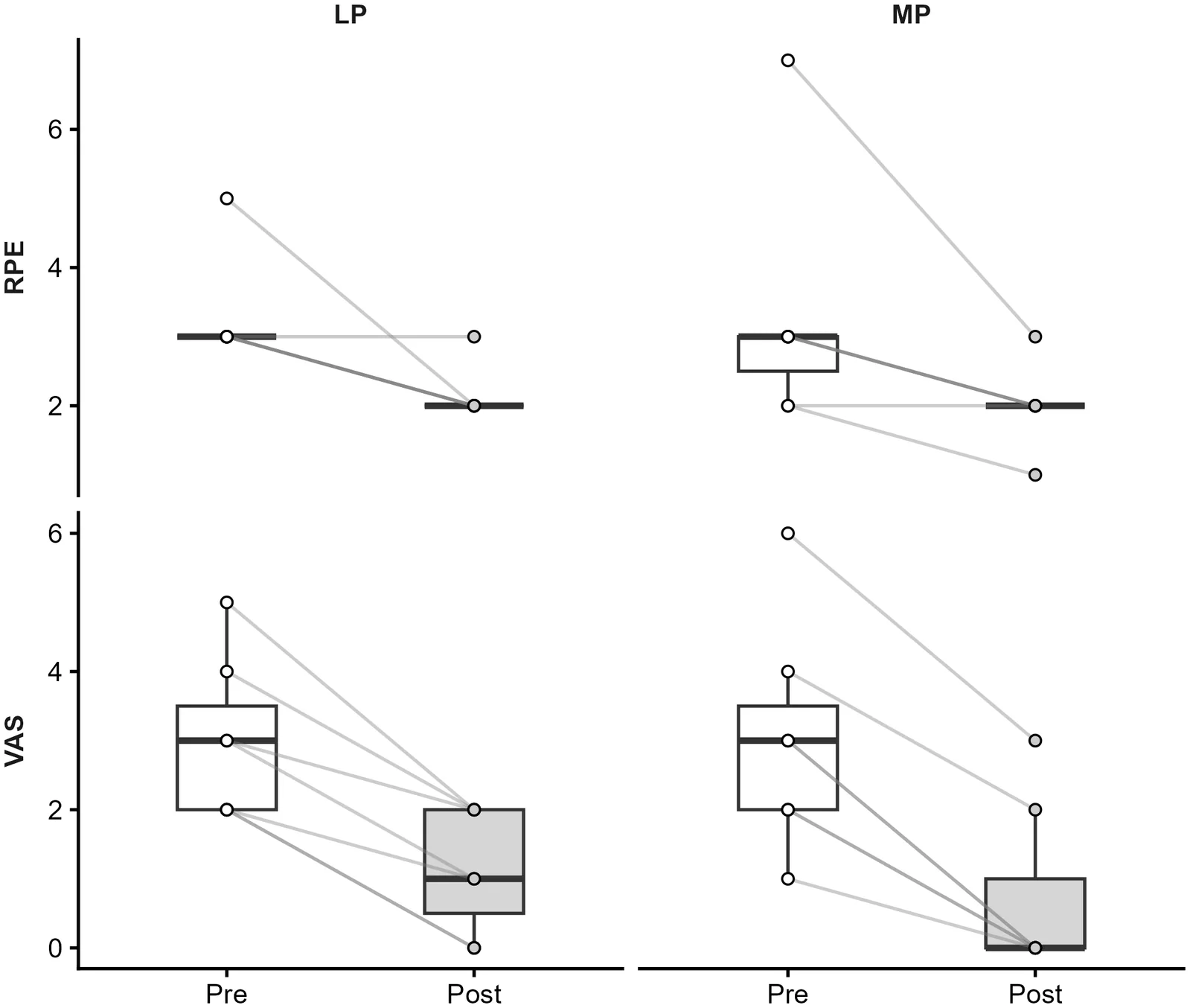
Subjective fatigue and pain ratings before and after intermittent cold compression. Note: The panels display ratings of perceived exertion (RPE) and visual analog scale (VAS) pain scores under low-pressure (LP) and moderate-pressure (MP) conditions. Open circles represent individual participants (n = 7), and connecting lines indicate paired pre-to-post changes. Boxplots represent the group distribution as the median and interquartile range. White and gray boxes indicate pre-intervention and post-intervention measurements, respectively.

## Discussion

The present study examined the effects of ICC at different pressure levels on the immediate recovery of the gastrocnemius muscle in collegiate soccer players. The results revealed significant post-intervention decreases in CMJ height and peak power, while subjective pain scores were significantly alleviated, consistent with previous findings [11] and suggesting a transient reduction in neuromuscular performance following a single 20-minute ICC session. Furthermore, the present study identified significant pressure × time interactions for RFD and the lateral gastrocnemius logarithmic decrement. These findings suggest that external mechanical pressure may contribute to differences in the acute neuromuscular and mechanical responses to ICC, potentially influencing the short-term recovery pattern of lower-limb explosive function.

### RFD and logarithmic decrement

Although peak force and peak power declined following ICC, the present study found that RFD and the lateral gastrocnemius logarithmic decrement did not exhibit a uniform response pattern but instead showed significant pressure × time interactions. The post hoc findings, however, differed between outcomes. For lateral gastrocnemius logarithmic decrement, a significant pre-to-post increase was observed under the LP condition, whereas no significant change occurred under the MP condition. In contrast, neither pressure condition showed a significant pre-to-post change in RFD. These findings suggest that compression pressure may influence the acute response pattern; however, given the small sample size, the results should be interpreted cautiously and require confirmation in larger studies. This may indicate that mechanical pressure contributes to the acute neuromuscular response to ICC in addition to the effects associated with cooling.

From a biomechanical perspective, RFD is constrained not only by the rate of neural drive recruitment but also by the stiffness and viscoelastic properties of the muscle-tendon complex [16]. In the present study, the lateral gastrocnemius logarithmic decrement showed different response patterns under the two pressure conditions, alongside the pressure × time interaction observed for RFD. Previous research has indicated that tissue cooling significantly increases muscle viscosity and dynamic stiffness, leading to greater internal resistance during contraction [17]. The differing response observed under the MP condition may indicate a different response pattern to these cooling-related mechanical changes. We hypothesize that moderate mechanical pressure may provide support and stabilization to muscle fibers, reducing non-productive deformation during the initial phase of contraction and thereby enabling more direct and efficient force transmission [18,19]. Furthermore, Previous studies have suggested that external compression may influence interstitial pressure and local fluid movement [17,20,21], which could potentially modify the mechanical environment of the muscle during cooling. Through this combination of mechanical stabilization and improved internal circulation, muscle fibers may transmit tension more efficiently during rapid contraction. These mechanisms may partly explain the different RFD response patterns observed between the two pressure conditions, although the within-condition changes in RFD did not reach statistical significance. Collectively, these findings suggest that compression pressure may influence the acute mechanical response of muscle during ICC, although the underlying mechanisms and clinical relevance require further investigation.

### Explosive power

The present study found that a single ICC session led to an overall decline in lower limb explosive power. Regardless of whether LP or MP was applied, CMJ height, peak force, and peak power were significantly reduced compared to pre-intervention values. This finding is consistent with the systematic review by Bleakley et al. [22], indicating that cryotherapy typically acutely impairs vertical jump and sprint performance, exerting a negative impact on neuromuscular explosive output. The acute reduction in CMJ performance may be consistent with previously reported effects of local cooling on neural and contractile processes. Previous studies have shown that cooling can reduce nerve conduction velocity and alter muscle spindle sensitivity [23,24], while reductions in muscle temperature may decrease myosin ATPase activity [25]. These mechanisms may contribute to transient reductions in explosive performance. However, nerve conduction, muscle temperature, muscle spindle activity, and myosin ATPase activity were not directly assessed in the present study; therefore, these mechanisms remain literature-based hypotheses rather than mechanisms demonstrated by the present data.

### Muscle architecture

In contrast to the significant changes observed in motor function and mechanical properties, a single ICC session did not induce significant changes in gastrocnemius muscle thickness or pennation angle (*p*>0.05). This finding aligns with established physiological principles of adaptation: remodeling of muscle fascicle architecture typically requires cumulative mechanical loading over extended periods, and a single, short-duration ICC intervention is unlikely to directly alter macroscopic muscle morphology [26].

Previous research has suggested that short-term fluctuations in muscle dimensions may reflect edema-related muscle swelling or interstitial fluid redistribution rather than structural hypertrophy [27]. However, no significant pressure-dependent changes in gastrocnemius muscle thickness or pennation angle were observed in the present study. Accordingly, the current findings do not provide sufficient evidence to infer differential effects of LP and MP on interstitial fluid clearance or decongestion. Future studies incorporating direct assessments of local fluid dynamics or edema-related outcomes are needed to determine whether compression pressure influences interstitial fluid redistribution during ICC.

### Subjective pain and fatigue

The present study demonstrated that ICC significantly improved post-exercise subjective perceptions. Regardless of whether LP or MP was applied, both pain scores and fatigue ratings decreased significantly following the intervention. This finding is consistent with previous research [28], with the analgesic effect primarily attributable to the synergistic action of cold stimulation and mechanical pressure. Local cooling reduces the conduction velocity of peripheral nociceptive nerve fibers, including A-delta and C fibers, and suppresses the release of local inflammatory mediators, thereby elevating the pain threshold [29]. Concurrently, external pressure stimulates large-diameter sensory nerve fibers, blocking the transmission of pain signals at the spinal level in accordance with the gate control theory [30]. Additionally, intermittent compression promotes venous return, alleviating tissue tension caused by local edema [31].

From a statistical perspective, no significant pressure × time interactions were observed for either subjective measure (*p*>0.05), providing no evidence of a differential response between the LP and MP conditions. Accordingly, the present data do not support an additional subjective benefit of MP over LP. Whether lower compression pressure is sufficient to achieve comparable perceptual effects should be examined in larger studies.

### Limitations and future directions

The present study has several limitations. (1) The sample size was relatively small, with only seven participants included. Although the sample size met the minimum requirement determined by the a priori power analysis and the randomized crossover design reduced between-participant variability, the absolute sample size remains limited, particularly given the number of physiological and biomechanical outcomes examined. Therefore, the estimates should be interpreted cautiously, and larger, adequately powered studies are required to confirm the present findings. In addition, although all participants reported high perceived exertion immediately after the 90-minute soccer-specific exercise protocol (Borg CR10 RPE ≥8), no objective functional or physiological measure was obtained both before and after the exercise protocol to independently quantify the magnitude of fatigue induced. Therefore, the post-exercise RPE values support substantial perceived exertion but do not provide an objective measure of the individual fatigue response. (2) A minimum 48-hour washout period was implemented between the two crossover sessions to reduce the potential for carryover effects. Although the participants were trained collegiate soccer players accustomed to regular high-intensity soccer training, complete recovery before the subsequent experimental session was not objectively verified using pre-exercise biochemical or functional markers. Therefore, residual fatigue or muscle damage from the preceding session cannot be entirely excluded and may have influenced the subsequent responses. Future crossover studies should consider longer recovery intervals and incorporate objective pre-session recovery assessments to confirm restoration of baseline status. (3) The study examined only the immediate response following a single intervention session. While this provides directly applicable information for designing acute post-match recovery protocols, the findings cannot be directly extrapolated to long-term physiological adaptation patterns. Future longitudinal research should therefore examine the effects of repeated ICC exposure on longer-term neuromuscular and morphological adaptations. (4) Biochemical markers related to exercise-induced muscle damage, inflammation, or metabolic recovery were not assessed in the present study. Therefore, the current findings are limited to mechanical, morphological, functional, and perceptual responses and cannot determine whether ICC or different compression pressures influenced the underlying biochemical recovery processes. Previous research has shown that biomarker responses in soccer may vary according to exercise modality and playing position; for example, Slimani et al. reported modality- and position-specific changes in metabolic, protein, inflammatory, and lipid-related biomarkers when comparing competitive match play with the VAMEVAL test in elite female soccer players [32]. These findings highlight the value of integrating biochemical monitoring with biomechanical and functional assessments in future ICC studies. (5) The present study relied primarily on laboratory-based assessments conducted at discrete time points and did not incorporate longitudinal field-based monitoring of recovery status. Previous research has shown that multimodal monitoring approaches integrating physiological measures, prior exercise characteristics, and self-perceived condition may be responsive to changes in post-exercise recovery status [33]. Although these findings were obtained in a different athletic population, they highlight the potential value of complementing laboratory-based biomechanical and functional assessments with repeated external monitoring. Future studies should therefore combine laboratory assessments with repeated field-based monitoring to better characterize recovery trajectories following ICC. (6) the present study did not include a passive-recovery, sham, or cooling-only control condition. Therefore, pre-to-post changes common to both ICC conditions cannot be distinguished from spontaneous recovery occurring over the same time interval, and the independent contributions of cooling and compression cannot be determined. Future studies should incorporate appropriate passive-recovery, sham, and/or cooling-only conditions to isolate these effects. Additionally, the present study compared only the effects of different pressure levels. Future research should explore various combinations of pressure, temperature, and intervention duration to develop more precise and individualized recovery strategies for sports practice.

## Conclusions

This study compared the immediate recovery effects of ICC at different pressure levels on the gastrocnemius muscle in collegiate soccer players. The results demonstrated that both pressure conditions were associated with reductions in subjective pain and RPE scores, while CMJ height, peak force, and peak power declined following ICC. Significant pressure × time interactions were observed for lateral gastrocnemius logarithmic decrement and RFD, suggesting that compression pressure may influence the acute mechanical and neuromuscular responses to ICC. However, the post hoc findings differed between outcomes, and neither pressure condition showed a significant pre-to-post change in RFD. Therefore, the interaction findings should be interpreted cautiously, particularly given the small sample size. These findings suggest that compression pressure may be an important factor influencing the acute response to ICC. Further studies with larger samples are warranted to determine the optimal pressure parameters for specific recovery goals.

## Supporting information

S1 File Participant-level data underlying the analyses reported in the manuscript. (XLSX)

S2 File Descriptive statistics for all study outcomes. (XLSX)

## Abbreviations

ICC: Intermittent Cold Compression
LP: Low Pressure
MP: Moderate Pressure
CMJ: Countermovement Jump
RFD: Rate of Force Development
VAS: Visual Analog Scale
RPE: Rating of Perceived Exertion
ANOVA: Analysis of Variance
